# The Reversed Halo Sign: A Rare Presentation of Legionella Pneumonia

**DOI:** 10.1590/0037-8682-0565-2025

**Published:** 2026-02-16

**Authors:** Alexandre Dias Mançano, Gláucia Zanetti, Edson Marchiori

**Affiliations:** 1 University of Florida, Department of Radiology, Gainesville, Florida, USA.; 2 Universidade Federal do Rio de Janeiro, Departamento de Radiologia, Rio de Janeiro, RJ, Brasil.

A 28-year-old man presented with a 2-day history of fever (39ºC), myalgia, and dry cough, followed by general condition decline and onset of chills, left-sided pleuritic pain, and episodes of diarrhea. Physical examination revealed crackles in the lower third of the left lung. The patient’s oxygen saturation in ambient air was 97%.

**FIGURE 1:**
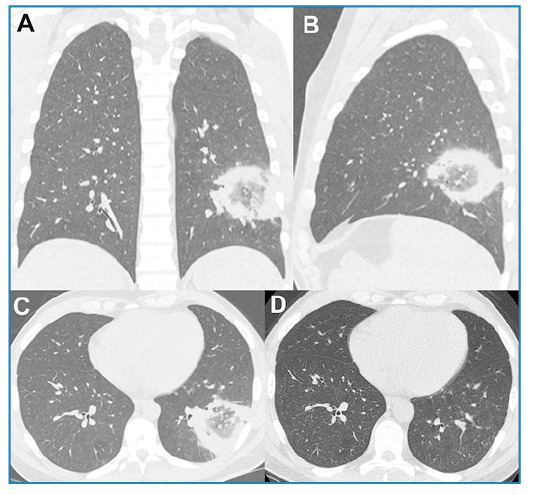
Chest computed tomography (CT) images with coronal **(A)**, sagittal **(B),** and axial **(C)** reconstruction showing a rounded area of ground-glass opacity surrounded by a complete ring of consolidation in the left lower lobe, compatible with the reversed halo sign. Control CT performed three weeks after the initial examination **(D)** revealed complete lesion regression.

Laboratory tests revealed leukocytosis, elevated C-reactive protein level (26.3 mg/L), and hyponatremia (128 mmol/L; reference range, 135-145 mmol/L). Human immunodeficiency virus (HIV) testing and blood cultures were negative. Chest computed tomography (CT) revealed a reversed halo sign (RHS) in the left lower lobe (Figure 1A-C ). A polymerase chain reaction panel for respiratory infections tested positive for *Legionella pneumophila*.

The urinary antigen test result for *Legionella* was also positive. Bronchoalveolar lavage cultures confirmed the presence of *L. pneumophila*. The final diagnosis of *L. pneumophila* pneumonia was established. Azithromycin (500 mg for 14 days) was prescribed and yielded an excellent clinical response. Three weeks after the initial examination, a control CT revealed complete regression of the lesion (Figure 1D).

*L. pneumophila* is an aerobic gram-negative bacillus. The most common CT findings are consolidation and/or ground-glass opacities. Pleural effusion was frequently observed. Cavitation and lymphadenopathy are common in immunocompromised patients^1^ ^-^ ^3^. RHS is a focal, rounded area of ground-glass opacity surrounded by a complete or nearly complete ring of consolidation on chest CT. It was initially considered specific to cryptogenic organizing pneumonia, but has since been reported to be associated with diverse clinical entities, including infectious and noninfectious diseases^4^ ^,^ ^5^.
